# Supplementary material for: Spatially dispersing Yu-Shiba-Rusinov states in the unconventional superconductor FeTe0.55Se0.45
Source: Nat Commun. 2021 Jan 12;12:298. doi: 10.1038/s41467-020-20529-x (PMC7804303; doi:10.1038/s41467-020-20529-x)
Supplement: Supplementary file 1 — Supplementary Information [file 41467_2020_20529_MOESM1_ESM.pdf]

Supplementary Information: Spatially dispersing Yu-Shiba-Rusinov states in the unconventional superconductor $FeTe_{0.55}Se_{0.45}$

Damianos Chatzopoulos,[1,*] Doohee Cho,[1,2,*] Koen M. Bastiaans,[1,*] Gorm O. Steffensen,[3] Damian Bouwmeester,[1,4] Alireza Akbari,[5,6] Genda Gu,[7] Jens Paaske,[3] Brian M. Andersen,[3] and Milan P. Allan[1,†]

[1] *Leiden Institute of Physics, Leiden University, Niels Bohrweg 2, 2333 CA Leiden, The Netherlands*
[2] *Department of Physics, Yonsei University, Seoul 03722, Republic of Korea*
[3] *Center for Quantum Devices, Niels Bohr Institute, University of Copenhagen, Universitetsparken 5, 2100 Copenhagen Ø, Denmark.*
[4] *Kavli Institute of Nanoscience, Delft University of Technology, Lorentzweg 1, 2628 CJ Delft, Netherlands*
[5] *Max Planck Institute for the Chemical Physics of Solids, D-01187 Dresden, Germany*
[6] *Max Planck POSTECH Center for Complex Phase Materials, and Department of Physics, POSTECH, Pohang, Gyeongbuk 790-784, Korea*
[7] *Condensed Matter Physics and Materials Science Department, Brookhaven National Laboratory, Upton, NY 11973, USA*

SUPPLEMENTARY NOTE 1: DECONVOLUTION OF SPECTRAL DENSITY

In order to obtain the local density of states (LDOS) of the sample we use a deconvolution algorithm which subtracts the density of states of the tip from the measured spectra. We follow the same procedure as in Refs. [1, 2]. The first step is to characterize the tip density of states. This is done by taking spectra with a Pb tip on Pb as described elsewhere [3]. We fit these spectra with

$$\left(\frac{dI}{dV}\right)_{\mathrm{Pb-Pb}} = \frac{1}{eR_{\mathrm{N}}}\int\left\{\frac{\partial D_{\mathrm{t}}(\omega+eV)}{\partial V}\left[f(\omega)-f(\omega+eV)\right]-D_{\mathrm{t}}(\omega+eV)\frac{\partial f(\omega+eV)}{\partial V}\right\}D_{\mathrm{t}}(\omega)d\omega, \tag{1}$$

in order to extract the gap $\mathit{\Delta}_{\mathrm{t}}$ and the broadening term γ_{t} of the tip. For the $D_{\mathrm{t}}(\omega,\mathit{\Delta}_{\mathrm{t}},\gamma_{\mathrm{t}})$, a modified Dynes formula is used

$$D_{\mathrm{t}}(\omega,\mathit{\Delta}_{\mathrm{t}},\gamma_{\mathrm{t}}) = \mathrm{Re}\left[\mathrm{sgn}(\omega)\frac{\omega}{\sqrt{\omega^2+2i\gamma_{\mathrm{t}}\omega-\mathit{\Delta}_{\mathrm{t}}^2}}\right]. \tag{2}$$

Good agreement is found for $\mathit{\Delta}_{\mathrm{t}} = 1.3$ meV and $\gamma_{\mathrm{t}} = 45$ μeV. Next, we discretize the theoretical tunneling formula for the differential conductance

$$\left(\frac{dI}{dV}\right)_j = \frac{1}{eR_{\mathrm{N}}}\sum_i\left\{\frac{\partial D_{\mathrm{t}}(\omega_i+eV_j)}{\partial V_j}\left[f(\omega_i)-f(\omega_i+eV_j)\right]-D_{\mathrm{t}}(\omega_i+eV_j)\frac{\partial f(\omega_i+eVj)}{\partial Vj}\right\}\delta\omega D_{\mathrm{s}}(\omega_i), \tag{3}$$

where $\delta\omega$ is the energy spacing. Note that we dropped the $\mathit{\Delta}_{\mathrm{t}}, \gamma_{\mathrm{t}}$ and T dependence for simplicity. The above formula is then solved in a matrix form in order to obtain $D_{\mathrm{s}}(\omega)$.

SUPPLEMENTARY NOTE 2: ANDERSON IMPURITY IN A (ZERO-BANDWIDTH) BCS SUPERCONDUCTOR

We model the sub-surface impurity by an Anderson impurity embedded in a simple s-wave superconductor. The total Hamiltonian reads:

$$H_{\mathrm{tot}} = H_{\mathrm{S}} + H_{\mathrm{T}} + H_{\mathrm{IMP}}, \tag{4}$$

---

* These authors contributed equally to this work
† allan@physics.leidenuniv.nl

where H_S is the Hamiltonian for the superconductor, H_T the tunneling contribution and H_IMP describes the impurity. In terms of fermion creation and annihilation operators for the superconductor (c) and the impurity level (d), each term is given by

$$H_\mathrm{S} = \sum_{k,\sigma} \epsilon_k c^\dagger_{k\sigma} c_{k\sigma} + \sum_k (\mathit{\Delta}_\mathrm{s} c^\dagger_{k\uparrow} c^\dagger_{k\downarrow} + \mathit{\Delta}^*_\mathrm{s} c_{k\downarrow} c_{k\uparrow}), \tag{5}$$

where $\mathit{\Delta}_\mathrm{s}$ is the superconducting gap (here assumed to be real), and

$$H_\mathrm{T} = \sum_{k,\sigma} t_\mathrm{s} c^\dagger_{k,\sigma} d_\sigma + t^*_\mathrm{s} d^\dagger_\sigma c_{k,\sigma}, \tag{6}$$

where we take $t_\mathrm{s} = \sqrt{\mathit{\Gamma}_\mathrm{s}/\pi\nu_\mathrm{F}}$ and ν_F is the density of states near the Fermi energy for $\mathit{\Delta}_\mathrm{s} = 0$, which is taken to be constant.

$$H_\mathrm{IMP} = \sum_\sigma \epsilon_0 d^\dagger_\sigma d_\sigma + U d^\dagger_\uparrow d_\uparrow d^\dagger_\downarrow d_\downarrow, \tag{7}$$

with U being the charging energy of the impurity.

In the normal state, this reduces to the standard Anderson model, including mixed-valence as well as Kondo physics below a characteristic Kondo temperature, T_K. In the superconducting state, this simplifies to a single Yu-Shiba-Rusinov (YSR) state, which can be tuned through zero energy by varying ϵ_0, as long as $T_\mathrm{K} \lesssim 0.3\mathit{\Delta}_\mathrm{s}$ [4]. Here, we employ the zero-bandwidth approximation (ZBW), which replaces the superconducting by a single pair of BCS quasiparticles at the gap edge,

$$H^\mathrm{ZBW}_\mathrm{S} = \mathit{\Delta}_\mathrm{s} c^\dagger_\uparrow c^\dagger_\downarrow + \mathit{\Delta}^*_\mathrm{s} c_\downarrow c_\uparrow. \tag{8}$$

From comparison with numerical renormalization group calculations, this approximation is known to capture the YSR states very well up to adjustments in the tunnel coupling, the value of which is fitted anyway [5].

In order to calculate the spectral weights of the impurity resonances in the Anderson model we use the Lehmann spectral representation of the Green's function. Using the many-body eigenstates $|n\rangle$ and the eigenenergies E_n of the ZBW Hamiltonian, the retarded Green's function on the impurity can be calculated as follows:

$$G^\mathrm{R}_{\sigma\sigma'}(\omega) = \frac{1}{Z} \sum_{nn'} e^{-\beta E_n} \left(\frac{\langle n|d_\sigma|n'\rangle \langle n'|d^\dagger_{\sigma'}|n\rangle}{\omega + i\mathit{\Gamma}_\mathrm{r} + E_n + E_{n'}} + \frac{\langle n|d^\dagger_{\sigma'}|n'\rangle \langle n'|d_\sigma|n\rangle}{\omega + i\mathit{\Gamma}_\mathrm{r} + E_{n'} - E_n} \right), \tag{9}$$

where $\beta = 1/(k_\mathrm{B}T)$ and the partition function Z is given by $Z = \sum_n e^{-\beta E_n}$. Here we have included a phenomenological relaxation rate, $\mathit{\Gamma}_\mathrm{r}$, which endows the otherwise sharp bound states by a finite lifetime broadening. The details of this quasiparticle relaxation time are beyond the scope of this work, but our analysis of the tunnelling current assumes it to be larger than or similar to the tip-impurity tunnelling rate, $\mathit{\Gamma}_\mathrm{t}$. The local spectral function is expressed in terms of the impurity retarded Green's function as:

$$D_\mathrm{I}(\omega, \epsilon_0) = -\frac{1}{\pi} \mathrm{Im} \left[\sum_\sigma G^\mathrm{R}_{\sigma\sigma}(\omega) \right]. \tag{10}$$

Supplementary figs. 1 and 2 show how this local density of (YSR) states changes with level energy, ϵ_0, and tunnelling rate $\mathit{\Gamma}_\mathrm{s}$, respectively. The quantum phase transition is revealed as the point by which the YSR state crosses zero energy and the spectral weight is exchanged between positive, and negative energy states. This asymmetry in spectral weight has a simple origin within the ZBW model. The observed excitation is between a doublet state, which is simply a single electron on the dot ($|\uparrow_\mathrm{d}, 0_\mathrm{qp}\rangle$, $|\downarrow_\mathrm{d}, 0_\mathrm{qp}\rangle$), and a more complicated singlet state. This singlet state continuously evolves from an empty impurity level, $|0_\mathrm{d}, 0_\mathrm{qp}\rangle$, at $\epsilon_0/U \gg 0$ to a doubly occupied level, $|2_\mathrm{d}, 0_\mathrm{qp}\rangle$, at $\epsilon_0/U \ll -1$. In between, these two singlets are mixed with the YSR singlet, formed by the singly occupied impurity level and the single BCS quasiparticle doublet, i.e.$|\uparrow_\mathrm{d}, \downarrow_\mathrm{qp}\rangle - |\downarrow_\mathrm{d}, \uparrow_\mathrm{qp}\rangle$ states. As the positive (negative) part of the spectral function is related to excitations from adding an electron (hole) to the ground state, this part is largest for $\epsilon_0/U \gg 0$ ($\epsilon_0/U \ll -1$). Precisely at $\epsilon_0/U = -0.5$ the state is equally composed of empty and doubly occupied components and as such the spectrum shows no asymmetry. The larger $\mathit{\Gamma}_\mathrm{s}$, the larger the gate-range around $\epsilon_0/U = -0.5$ in which the YSR singlet component dominates this excited singlet state, and the lower its energy. This explains both the closing of the doublet sector and the increased spectral symmetry as $\mathit{\Gamma}_\mathrm{s}/\mathit{\Delta}_\mathrm{s}$ increases, observed in Supplementary fig. 1.

As is evident from these two figures, the phase transition may be induced either by tuning the level energy, ϵ_0, or the tunnel coupling, t_s. As explained in the main text, previous experiments [6] have reported a tip-induced tuning of t_s by assuming a force acting on the impurity from the tip. Whereas this seems feasible for a flexible molecule placed on the surface, we believe that this possibility is less likely in the present case, where the impurity is sub-surfactant. No indication of lattice deformation when tip-sample distance changes (pushing or pulling) is observed in our experiments. Therefore, we consider the effective gating mechanism much more likely to explain the observed tuning of the sub-gap states.

SUPPLEMENTARY NOTE 3: AZIMUTHALLY-AVERAGED RADIAL PROFILES AND SPATIAL MAPPING OF THE YSR RESONANCES

For completeness in Supplementary figs. 3a-e we present all the five azimuthally-averaged radial profiles, each at different tip-sample distance. In Supplementary fig. 3f we also show a spatial map of the YSR resonances obtained by fitting a lorentzian.

SUPPLEMENTARY NOTE 4: NOISE SPECTROSCOPY

As an extra verification for the performance of our junction, we used the Scanning Tunneling Noise Microscopy (STNM) technique [7, 8] to measure the current noise as function of energy (noise spectroscopy) on a single location on the $FeTe_{0.55}Se_{0.45}$ surface, not at an impurity location, with the aim to look for the doubling of current noise due to Andreev reflections. Supplementary fig. 4a shows the measured current noise power as function of applied bias $S(V)$, with the out of tunneling noise subtracted to remove the thermal noise component and input noise of the amplifier $S(V) - S(0)$. The dashed lines indicate the predicted shot noise curve [9–11] for tunneling of single electron charge (e) and double electron charge ($2e$). At a bias voltage larger than the superconducting gap energies, $eV > |\Delta_\mathrm{t} + \Delta_\mathrm{s}|$, the measured noise power data follows the predicted noise power for single electron tunneling. When the bias is lowered below the superconducting gap energies, $eV < |\Delta_\mathrm{t} + \Delta_\mathrm{s}|$, the current noise clearly deviates from single electron charge transfer, showing a doubling of noise power to the $2e$ line, consistent with the appearance of Andreev reflection processes [10–12]. To show the effective charge transferred between the Pb tip and $FeTe_{0.55}Se_{0.45}$ surface we divide the measured noise power by the full Poissonian noise for single electron charge transport $S = 2e|I|$, which is shown in Supplementary fig. 4b. This illustrates a clear step from e to $2e$ charge transfer when the bias is lowered below the superconducting gap energies $eV < |\Delta_\mathrm{t} + \Delta_\mathrm{s}|$, demonstrating that the tunneling current is now effectively carried by double charge quanta due to Andreev reflection processes that start to dominate. This is the first time such noise enhancement due to Andreev reflection processes is shown in a junction containing an unconventional superconducting electrode (the $FeTe_{0.55}Se_{0.45}$ surface), opening a potential new path for further investigation of the YSR or Majorana states by the means of noise spectroscopy.

SUPPLEMENTARY NOTE 5: ESTIMATION OF THE POTENTIAL DROP IN THE VACUUM BARRIER BETWEEN TIP AND SAMPLE

In an STM experiment there is a potential drop in the vacuum tunneling barrier [13] due to the work function difference between tip (W_t) and sample (W_s), as illustrated in Supplementary fig. 5a-c. As we explain in the main text, this potential drop results into an effective electric field penetration in the sample which acts as a local gate. An estimation of the work function difference yields that in our experiment the tip has a larger work function than the sample. In more detail, we are using a Pt/Ir tip coated with Pb. From literature we find that the work functions are $W_{\mathrm{Pt/Ir}} = 5 - 6$ eV and $W_{\mathrm{Pb}} = 4.25$ eV, respectively. This yields a rough estimation of the work function of the tip $W_{\mathrm{tip}} = 4.5 - 5$ eV. Concerning the top surface of our sample it consists of Se, $W_{\mathrm{Se}} = 5.9$ eV and Te, $W_{\mathrm{Te}} = 4.95$ eV atoms. However, previous STM experiments [14] show that Fe(Se,Te) has a significantly smaller work function of ~ 3 eV. This yields a work function difference of the order of 1 eV.

We note that a vertical movement of the tip has a stronger influence of the field at the impurity location than a horizontal movement because the radius of the apex of the tip is large compared to the tunneling distance, and because the electric field decays algebraically. This is illustrated in Supplementary fig. 5d. The radius of the apex of typical STM tips is 10-100 nm. While the exact radius is unknown, we estimate it to be around 20 nm as the tip has been indented in Pb.

SUPPLEMENTARY NOTE 6: ESTIMATION OF THE CHARGING ENERGY U OF THE IMPURITY AND THE POTENTIAL CHANGE ON IT DUE TO THE TIP Δd MOVEMENT

Since we do not know the specific nature of the impurity, we make a rough estimate of its charging energy U by approximating the impurity orbital by a sphere of radius R. If the sphere already contains a single electron, the energy for placing the next will then be given by $U = e^2/(8\pi\varepsilon_r\varepsilon_0 R)$, where ε_0 is the vacuum permittivity and ε_r the dielectric constant of the medium that the impurity is embedded. Assuming that $R = 1 - 3$ nm and $\varepsilon_r \sim 15$ [15] we find that $U \sim 15 - 50$ meV. Next we want to show that when we move the STM tip closer to sample, we induce a potential shift on the impurity (which we assume that resides halfway between the topmost and the subsequent layer) that is comparable to the charging energy U, indicating that the gating scenario is possible. However, we emphasize that $FeTe_{0.55}Se_{0.45}$ is an unconventional superconductor (e.g. low Fermi energy, inhomogeneous superfluid density). Hence, the simple calculation below for band-bending might not be applicable here and is therefore performed just for illustration purposes.

At first, we estimate the potential drop on the surface of the sample based on the image charges method which assumes that a spherical tip with radius r_t is in tunneling distance h from the sample. According to previous studies on semiconductors and correlated electron systems the voltage drop on the surface or band-bending potential V_BB is given by [13]

$$V_\mathrm{BB} = \frac{1}{1 + \varepsilon_r \frac{h}{r_\mathrm{t}}}(V_\mathrm{b} - W), \tag{11}$$

here V_b is the bias voltage and W the work function difference between tip and sample ($W \sim 1$ eV and $V_\mathrm{b} \sim 1$ meV in our experiments). Moreover, we chose $r_\mathrm{t} = 20$ nm and $\varepsilon_r = 15$ as explained before. As a last step, we employ the following exponential model to fit our conductance (G) versus Δd curve, for the estimation of the tip-sample distance h

$$G = G_0 e^{-2kh}. \tag{12}$$

It is found that for the Δd tip movement shown in Fig. 3 the minimum (maximum) tip-sample distance is 2.9 (5.1) Å. This gives a change in the band-bending potential $\Delta V_\mathrm{BB} \approx 0.1$ V on the surface.

Finally, the potential shift on the impurity is estimated by an exponential decay inside the bulk governed by the Thomas-Fermi screening length $\lambda_\mathrm{TF} = 0.5$ nm, as calculated in the main text. That is

$$\Delta V_\mathrm{imp} = \Delta V_\mathrm{BB} e^{-d_\mathrm{imp}/\lambda_\mathrm{TF}}. \tag{13}$$

For the impurity depth d_imp we choose 0.25 nm, resulting in $\Delta V = 60$ meV. From the simulations in Fig. 4 we found that when ε_0 changes by $\sim 0.1U = 2$ meV the YSR states disperse from the gap edge to zero-bias. Since our rough estimate shows that we can induce a larger change, we conclude that the gating scenario is possible.

SUPPLEMENTARY NOTE 7: STATISTICS

On one sample, we investigated 5 ring-shaped in-gap impurity features in a 45×45 nm^2 field-of-view. All of them showed a dispersion when changing Δd and Δr. The dispersion in Δd is different in different impurities; the estimated cross of the YSR states at the Fermi level varies and is mostly below $\Delta d = -0.7$ Å. We did not measure all 5 impurities for smaller tip-sample distances but we expect similar X-shaped profiles. We assume that the different dispersions are due to their location inside the crystal. We can imagine that an impurity that is deeper in the crystal would be less prone to the influence of the electric field of the tip. On a second sample we found 2 additional impurity features extending over ~ 4 nm. Note that we do not observe any impurities with robust zero-bias peaks that do not disperse.

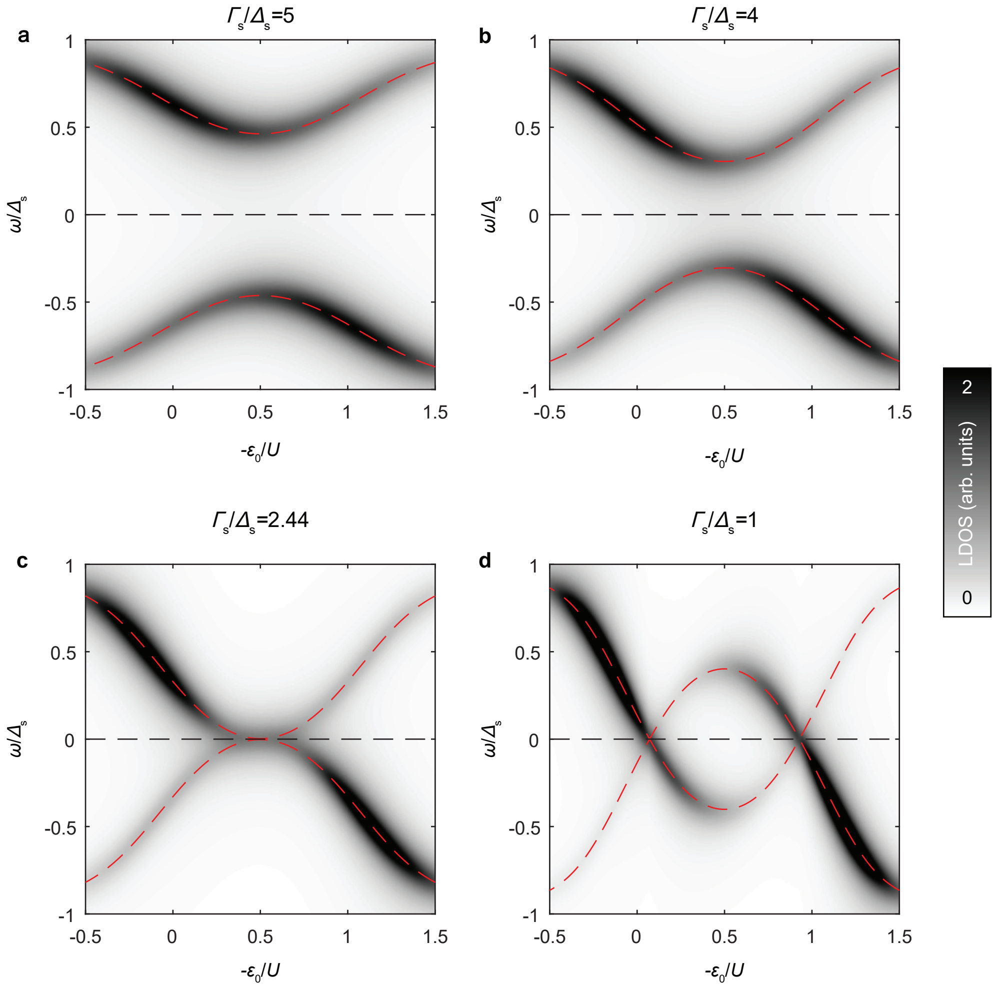

Supplementary Figure 1: Local density of (sub-gap) states as a function of energy (ω) and impurity level energy (ϵ_0). Different values of the tunneling rate Γ_s are indicated on the top of each panel. The (YSR) bound state energy is highlighted with red dashed lines. In all panels we used $U/\Delta_s = 3$, together with a phenomenological quasiparticle relaxation rate, $\Gamma_r = 0.1\Delta_s$.

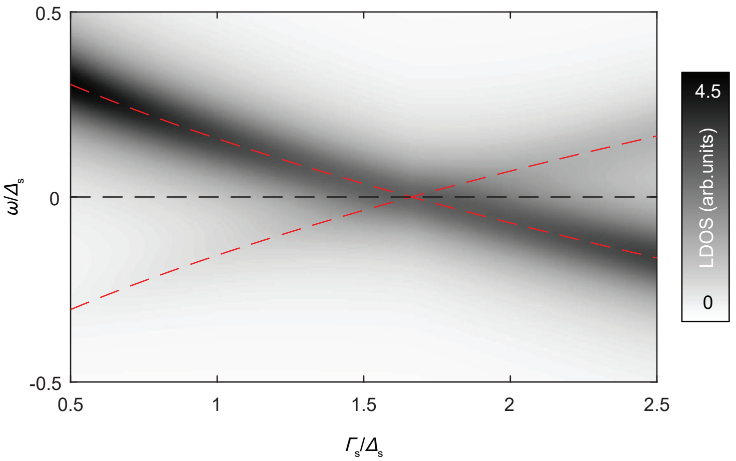

Supplementary Figure 2: Local density of (sub-gap) states as a function of energy (ω) and tunneling rate ($\mathit{\Gamma}_{\mathrm{s}}$). The (YSR) bound state energy is highlighted with red dashed lines. For the simulations we used $U/\mathit{\Delta}_{\mathrm{s}} = 3$, $\epsilon_0/\mathit{\Delta}_{\mathrm{s}} = -2.5$, together with a phenomenological quasiparticle relaxation rate $\mathit{\Gamma}_{\mathrm{r}} = 0.1\mathit{\Delta}_{\mathrm{s}}$.

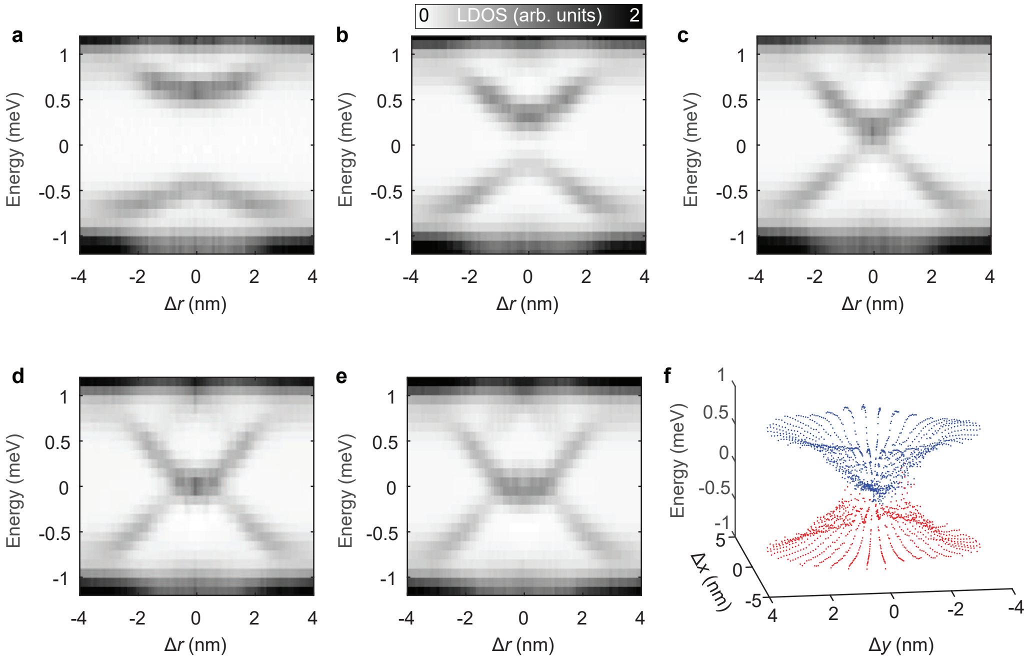

Supplementary Figure 3: Azimuthally-averaged radial profiles and spatial mapping of the YSR resonances. a-e Azimuthally-averaged radial cuts for $\Delta d(\text{Å}) = 1.1, -0.2, -0.7, -1.2$ and -1.4, respectively. Note that the colorbar applies to all panels. **f** Spatial mapping of the YSR energy resonances. The energy of each resonance is obtained by fitting a lorentzian at each point of $\text{LDOS}(\mathbf{r}, \omega)$ at $\Delta d(\text{Å}) = -1.2$. Blue (red) dots represent the positive (negative) resonances.

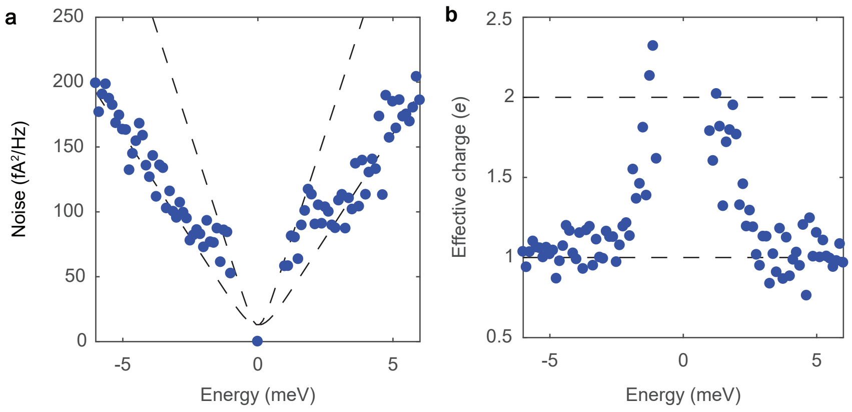

Supplementary Figure 4: Shot-noise measurement on $\mathrm{FeTe_{0.55}Se_{0.45}}$**. a** Measured current noise power (blue dots) as function of applied bias on the $\mathrm{FeTe_{0.55}Se_{0.45}}$ surface, while keeping a constant junction resistance of $R_{\mathrm{N}} = 10\ \mathrm{M\Omega}$. The black dashed lines represent the expected current noise power for $q = e$ and $q = 2e$ tunneling. **b** Effective charge (q) transferred between the Pb tip and $\mathrm{FeTe_{0.55}Se_{0.45}}$ surface tunnel junction, obtained by dividing the measured current noise power by the full poissonian noise $2e|I|$, similar to the Fano factor. Dashed lines indicate $q = e$ and $q = 2e$.

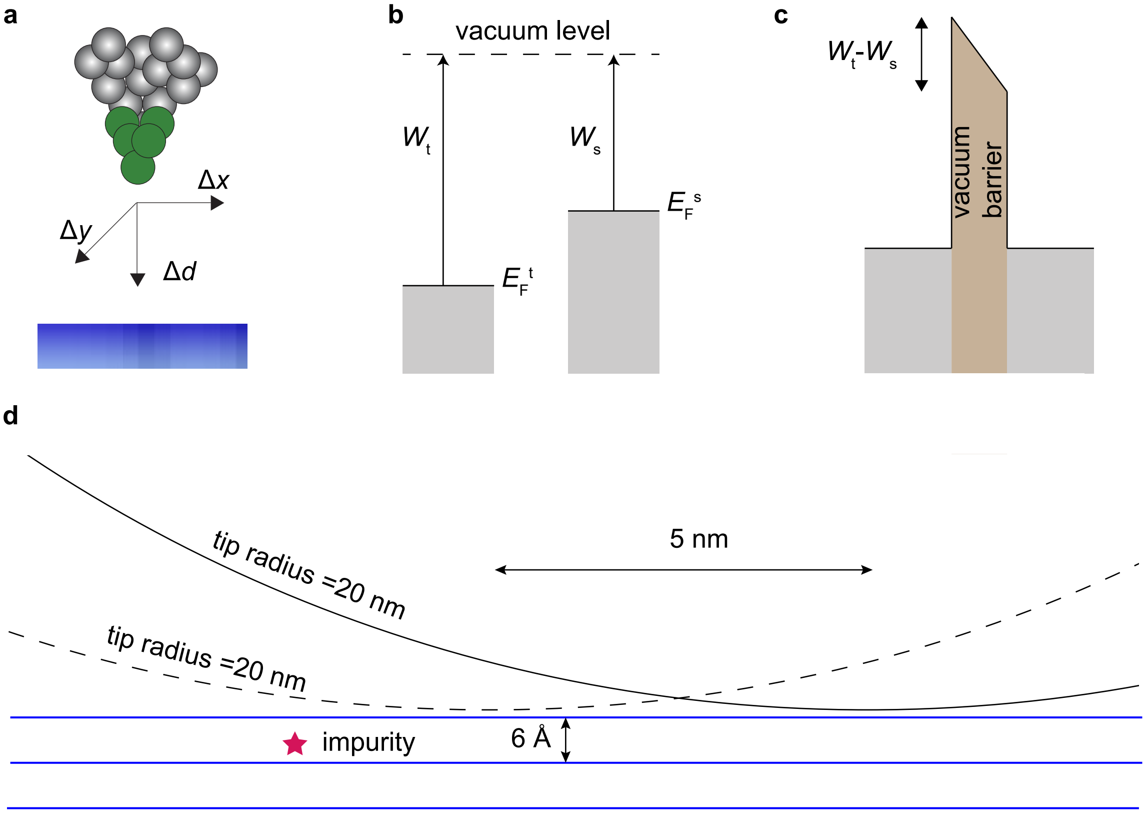

Supplementary Figure 5: Potential drop in the vacuum barrier in an STM junction and tip-shape illustration. **a** Schematic representation of the tip and sample. **b** Schematics of tip and sample density of states when they are isolated. Grey shaded areas indicate filled states whereas the work function is represented with arrows. **c** When a tunneling contact is formed due to the work function difference there is a potential drop in the vacuum barrier. **d** Tunneling junction schematics (in approximate scale). Two spherically shaped tips (radius is 20 nm) that are 5 nm apart are shown. An impurity resides bellow the surface (atomic planes are drawn as blue lines separated by 6 Å.

---

[1] Choi, D.-J. *et al.* Mapping the orbital structure of impurity bound states in a superconductor. *Nat. Commun.* **8**, 15175 (2017).

[2] Pillet, J.-D. *et al.* Andreev bound states in supercurrent-carrying carbon nanotubes revealed. *Nat. Phys.* **6**, 965–969 (2010).

[3] Cho, D., Bastiaans, K. M., Chatzopoulos, D., Gu, G. D. & Allan, M. P. A strongly inhomogeneous superfluid in an iron-based superconductor. *Nature* **571**, 541 (2019).

[4] Bauer, J., Oguri, A. & Hewson, A. C. Spectral properties of locally correlated electrons in a Bardeen–Cooper–Schrieffer superconductor. *J. Phys.: Condens. Matter* **19**, 486211 (2007).

[5] Grove-Rasmussen, K. *et al.* Yu-Shiba-Rusinov screening of spins in double quantum dots. *Nat. Commun.* **9**, 2376 (2018).

[6] Farinacci, L. *et al.* Tuning the Coupling of an Individual Magnetic Impurity to a Superconductor: Quantum Phase Transition and Transport. *Phys. Rev. Lett.* **121**, 196803 (2018).

[7] Bastiaans, K. M. *et al.* Amplifier for scanning tunneling microscopy at MHz frequencies. *Rev. Sci. Instrum.* **89**, 093709 (2018).

[8] Bastiaans, K. M. *et al.* Charge trapping and super-Poissonian noise centres in a cuprate superconductor. *Nat. Phys.* **14**, 1183–1187 (2018).

[9] Blanter, Ya. M. & Büttiker, M. Shot noise in mesoscopic conductors. *Phys. Rep.* **336**, 1 – 166 (2000).

[10] de Jong, M. J. M. & Beenakker, C. W. J. Doubled shot noise in disordered normal-metal–superconductor junctions. *Phys. Rev. B* **49**, 16070–16073 (1994).

[11] Cuevas, J. C., Martín-Rodero, A. & Yeyati, A. L. Shot Noise and Coherent Multiple Charge Transfer in Superconducting Quantum Point Contacts. *Phys. Rev. Lett.* **82**, 4086–4089 (1999).

[12] Bastiaans, K. M. *et al.* Imaging doubled shot noise in a Josephson scanning tunneling microscope. *Phys. Rev. B* **100**, 104506 (2019).

[13] Battisti, I. *et al.* Poor electronic screening in lightly doped Mott insulators observed with scanning tunneling microscopy. *Phys. Rev. B* **95**, 235141 (2017).

[14] Massee, F. A Tunneler's View on Correlated Oxides and Iron Based Superconductors, PhD thesis (2011).

[15] Zhao, W. *et al.* Direct imaging of electron transfer and its influence on superconducting pairing at FeSe/SrTiO3 interface. *Sci. Adv.* **4**, eaao2682 (2018).
